# Amorphous Carbon Film as a Corrosion Mitigation Strategy for Stainless Steel in Molten Carbonate Salts for Thermal Energy Storage Applications

**DOI:** 10.3390/ma17225619

**Published:** 2024-11-18

**Authors:** Miguel Morales, Mohammad Rezayat, Antonio Mateo

**Affiliations:** 1 CIEFMA-Department of Materials Science and Engineering, Universitat Politècnica de Catalunya, Barcelona-Tech, Campus Diagonal Besòs-EEBE, 08019 Barcelona, Spain; mohammad.rezayat@upc.edu (M.R.); antonio.manuel.mateo@upc.edu (A.M.); 2 Centre for Research in Multiscale Science and Engineering of Barcelona, Universitat Politècnica de Catalunya, Barcelona-Tech, Campus Diagonal Besós-EEBE, 08019 Barcelona, Spain

**Keywords:** molten carbonate salts, corrosion, stainless steel, thermal energy storage (TES), concentrated solar power (CSP), amorphous carbon film

## Abstract

Ternary carbonate salts (Li_2_CO_3_-Na_2_CO_3_-K_2_CO_3_) are promising heat transfer fluids to increase the efficiency of the electric power in concentrated solar power (CSP) technology. However, the corrosion produced at high operating temperatures is a key challenge to tackle for employing cost-effective steels as construction materials in CSP. In this work, the use of stainless steels with amorphous carbon was investigated, for the first time, as a surface modification method to mitigate the corrosion of structural CSP materials by molten salts. In doing so, an amorphous carbon (a-C) film of 100 nm in thickness was deposited on the 301LN stainless steel’s surface by the carbon thread evaporation technique. The corrosion behavior of the 301LN was assessed in carbonate salt at 600 °C for 1000 h. This film decomposed forming carbide layers, contributing to corrosion mitigation due to the generation of denser oxide layers, decreasing the Li^+^ diffusion through the stainless steel.

## 1. Introduction

Energy storage is crucial for a reliable renewable energy supply when sunlight and wind are unavailable. It enhances a grid’s flexibility, reliability, and power quality, supporting the growth of renewable energy. In particular, thermal energy storage (TES) technology offers many benefits, such as high efficiency, low operating costs, and scalability [1,2,3,4,5]. TES generally includes a storage medium, heat transfer system, and containment, and is compatible with concentrated solar power (CSP) plants [6,7,8]. Commercial CSP plants mostly use nitrate molten salts [9,10,11], while future generations of CSP technology are looking at carbonate and chloride salts for better high-temperature performance [12,13]. Carbonate salts have superior thermophysical properties and less corrosion than chlorides when interacting with materials like stainless steel [14,15,16,17]. However, carbonate salts present several challenges such as high melting temperatures (around 400 °C) and elevated costs [18,19,20]. Among these, eutectic LiNaK carbonate salt (32.1 wt.% Li_2_CO_3_; 33.4 wt.% Na_2_CO_3_; and 34.5 wt.% K_2_CO_3_) is the most efficient option [21,22].

Concerning the chemical compatibility of the containment materials and molten carbonate salts, many works have reported the corrosion resistance of austenitic stainless steels, such as AISI 310 and AISI 347 [22], AISI 316 [23], AISI 304 [24], duplex stainless steel 2205 [23,25,26], and Ni-base alloys, such as Fe-Cr-Al alloy (Kanthal), Haynes 214 and Haynes 230 [22], Inconel 601 [27], and HR3C [28], immersed in molten carbonate salts. Austenitic and duplex stainless steels in eutectic carbonate salts at 600–700 °C presented a multilayer of Li-mixed oxides as corrosion products, which are crucial for reducing the corrosion of these metallic alloys [22,23,24,27,29,30,31]. However, these studies evidence high corrosion rates, and CSP plants require containment materials with a lifetime of several decades [21,22]. Several effective strategies have recently been reported that use carbonate and nitrate salts, as follows: (1) nanofluids based on doping molten salts with nanoparticles [31], (2) surface modification of stainless steels through laser-texturing [32,33,34], and (3) protective coatings [35,36,37,38] through fractal-texturing [39,40,41,42]. Carbonate molten salt-based nanofluids, formed by 1 wt.% Al_2_O_3_ or SiO_2_ nanoparticles [43], CaO and MgO nanoparticles [44], or ZnO nanoparticles [45], can considerably improve the corrosion resistance of stainless steels and also enhance their thermophysical properties [31,46,47,48,49,50].

On the other hand, more recently, laser texturing has been proposed as an effective surface modification method for stainless steels to reduce their corrosion in high-temperature molten-salt applications. In this regard, Morales et al. [33], Rezayat et al. [34], and Grosu et al. [32,51] recently reported on LST as a promising approach for corrosion inhibition of AISI 310, AISI 301LN, and duplex steel 2205, respectively, in molten carbonate salts. Laser treatment caused the adhesion of hydrocarbon groups, which decomposed into carbon during the corrosion process. Carbonization by laser-texturing and the generation of metal–carbide layers stimulated the formation of denser protective corrosion layers mainly based on Cr, Fe, and Ni. Decreases in the Li and Cr diffusions during the corrosion tests were observed. Therefore, the corrosion rates of the stainless steels AISI 301LN, AISI 310, and duplex 2205 in molten salt decreased by 20% [34], 45% [51], and 48% [33] compared with the untreated ones, respectively.

Protective coatings as an anti-corrosion method has also been explored. Agüero et al. [52] used Al slurry coatings to protect ferritic/martensitic steels (P91, VM12, and MarBN) in solar salt at 550 °C and molten carbonate salts at 650 °C. The formation of NaAlO_2_ enhanced the protective characteristics of these coatings. Encinas-Sánchez et al. [53] proposed a sol-gel ZrO_2_-Y_2_O_3_ coating (with a thickness of 1.0–1.4 μm) deposited on P91 (a steel with low Cr-MoNi alloy content (P91)) in solar salt at 500 °C, which exhibited behavior similar to that of AISI 304. For molten chloride salts, Porcayo-Calderon et al. [54] concluded that Ni20Cr coatings display better performance than AISI 304 in ZnCl_2_-KCl molten salts at 350, 400, and 450 °C due to their high Ni content. Gomez-Vidal et al. [55] proposed the surface passivation through pre-oxidation treatments of Haynes 224, Inconel 702, and Kanthal APMT using alumina-forming alloys. The corrosion tests showed that the samples with alumina layers in molten chloride salts were unstable under an argon atmosphere and stable under an air one. In another work, Gomez-Vidal [56] decreased the corrosion rate to 0.190 mm/yr in molten chloride salts at 700 °C using atmospheric plasma spray NiCoCrAlY coatings pre-oxidized in air at 900 °C. On the other hand, Kondaiah and Pitchumani [40] reported fractal-textured Ni coatings on AISI 310, 316, and 347 and In800H, which were fabricated combining a chemical etching and electrodeposition technique, for corrosion mitigation in molten carbonate salts at 750 °C. They also studied the effect of fractal-textured Ni coatings in anhydrous molten chloride salts at 750 °C. The Ni-coated ferrous alloys reduced the corrosion rate by 70% over durations of 500 h long. In addition, they exhibited the same corrosion rate in industrial-grade AC salt after purification. Grosu et al. suggested spray-graphitization as an effective anti-corrosion treatment for carbon steel in molten HitecXL salt [35], HitecXL salt-based nanofluid [57], solar salt [58], and carbonate salts [36]. In molten carbonate salts at 600 °C, graphitization improved the compatibility of AISI 310 and AISI 347 due to the carbonate/carbide formation, which prevented chromium oxide dissolution and peel-off. The reaction of graphite with AISI 310 resulted in carbide formation, whereas the oxidation of graphite for AISI 347 resulted in carbonate formation.

This work presents a proof-of-concept study to assess amorphous carbon-coated stainless steel (AISI 301LN) as a new approach for corrosion mitigation in molten carbonate salt. The anti-corrosive properties of amorphous carbon films on stainless steels in aggressive corrosive environments, such as acid and alkaline solutions [59] and sea water [60], have been previously reported. Here, the amorphous carbon deposited onto the surface of the stainless steel was transformed into iron carbide during the corrosion test, enhancing the protective behavior of the oxide scales. This approach may be of interest in mitigating corrosion in stainless-steel components for CSPs, storage plants, molten-carbonate fuel cells, power-to-heat-to-power, etc., which undergo significant corrosion when in contact with carbonate salts at high temperatures.

## 2. Experimental Procedure

### 2.1. Materials

AISI 301LN stainless steel was provided by Outokumpu (Helsinki, Finland), which is referred to as 301LN in this document. The nominal chemical composition of the 301LN was bal. % Fe, 17.6% Cr, 6.5% Ni, 1.13% Mn, 0.02% C, and 0.17% N. Samples of 50 × 20 × 2 mm^3^ were used in the following two types of initial state: pristine steel and coated with amorphous carbon. The surfaces of all samples were ground with SiC grinding paper (1200 grit), cleaned with ethanol in an ultrasonic bath, and weighed on an analytical balance.

The coated samples were prepared by carbon thread evaporation (Leica ACE600 high-vacuum coater, Wetzlar, Germany) over the clean surfaces of one side of the stainless-steel plates, because for a hypothetical application, only one side would be in contact with the carbonate salts. During the deposition, the pressure in the chamber was 5 × 10^−4^ mbar, the evaporator voltage and current were 5 V and 40 A, respectively, at a 100 mm working distance. Single pulses of 150 ms were applied for a deposition time adjusted to achieve a film thickness of 100 nm, allowing the overall process to be completed in less than 10 min.

Eutectic molten carbonate salt, formed with 32 wt.% Li_2_CO_3_—33 wt.% Na_2_CO_3_—35 wt.% K_2_CO_3_, was prepared using a dry method, following previous works [36,37]. The chemical precursors, with a purity >99%, were provided by Sigma-Aldrich (St. Louis, MO, USA).

### 2.2. Corrosion Tests

For the corrosion tests, the samples were immersed in the molten salts in alumina crucibles in an air atmosphere at 600 °C. When the corrosion test finished, the samples were extracted from the molten salts and washed with deionized water. Corrosion rates were calculated from the loss of metal mass using the descaling method following ASTM-G1-03 [61]. The corrosion products formed on the 301LN substrates were removed using an aqueous solution with 10% HCl (*v*/*v*). From the mass loss measurements, the corrosion rate (*CR*) was calculated as follows:

(1) $$CR=\frac{8760\cdot ∆m}{\rho \cdot t}$$

where Δ*m* is the mass change per unit of initial surface area (mg/cm^2^), *ρ* is the density of the material (g/cm^3^), *t* is the exposure time (h), and 8760 is the number of hours per year. Each value of the mass change for each exposure time was obtained from at least three samples. Additional details of the experimental procedure can be found in a previous work [62].

### 2.3. Characterization of Amorphous Carbon Film

The morphological structure of the amorphous carbon layer was tested using a field emission scanning electron microscope (Carl Zeiss Merlin FESEM, Oberkochen, Germany) equipped with energy-dispersive spectroscopy (EDS) (Oxford Instruments INCA-350 system, Abingdon, UK). To observe the microstructure of the amorphous carbon film, cross-sections of samples were prepared using a focused ion beam (Carl Zeiss Neon 40 FIB, Oberkochen, Germany). The amorphization degree of the carbon coating was characterized by Raman spectroscopy (inVia Qontor, Renishaw, Wotton-Under-Edge, Gloucestershire, UK) [4,8,26,27]. A laser with an excitation wavelength of 514 nm at a low irradiation intensity was employed to minimize heating of the sample. The chemical composition of the amorphous carbon film was analyzed by X-ray photoelectron spectroscopy (XPS) with a SPECS system (Surface Nano Analysis GmbH, Berlin, Germany). The functional groups on the coated surfaces were qualitatively determined using an FTIR spectrometer [(Nicolet 6700, Thermo Scientific, Waltham, MA, USA)] equipped with CsI beam splitter, which was used in the spectral range of 6400–200 cm. The measurements were carried out in the reflectance mode and several accessories for measuring in the transmission, grazing-angle reflection, and attenuated total reflection modes.

### 2.4. Characterization of Oxide Scales

After the corrosion test, oxides scales of coated and non-coated samples were characterized. First, phase identification of the oxide scales was analyzed by X-ray diffraction (XRD, Bruker, D8-Advance, Billerica, MA, USA) using Cu Kα radiation (operated at 40 kV and 40 mA). The microstructures of the oxide scales on the top and cross-section surfaces of the samples were analyzed by an FESEM equipped with EDS (Carl Zeiss Merlin, Oberkochen, Germany). EDS was performed for elemental mapping. The cross-sections of the samples were embedded, ground, and polished, following a previous work [33]. To analyze the effect of amorphous carbon film on the mechanical properties of oxide scales, the hardness of the 301LN and the oxide scales for each condition (as-received, coated, and non-coated samples) were determined employing a Nanoindenter with a continuous stiffness measurement module (Nanoindenter XP System, Agilent Technologies, Santa Clara, CA, USA). A Berkovich diamond tip was used, which was calibrated with a fused silica standard. The hardness (*H*) was obtained using the Oliver and Pharr method [63]. The as-received and coated samples were embedded, ground, and polished following a previous work [62]. After corrosion tests, the amounts of minor elements in the carbonate salts were determined by the inductively coupled plasma optical emission spectroscopy technique [(Agilent Technologies 5100 ICP-OES, Santa Clara, CA, USA)]. Additional details on the experimental procedure can be found in a previous work [33].

## 3. Results and Discussion

### 3.1. Analysis of Amorphous Carbon Film

**Figure 1:**
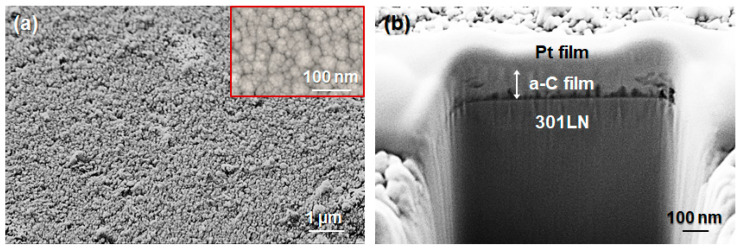
(**a**) Top-surface and (**b**) cross-sectional FIB-FESEM images of the a-C film deposited on the 301LN stainless-steel substrate.

After deposition of the a-C film, its chemical composition, microstructure, and structural properties were analyzed in detail. Figure 1 shows images obtained by FIB-FESEM on the top surface and cross-section of the a-C film deposited on the 301LN substrate. In Figure 1a, the top-surface view evidences that the a-C film was compact and homogeneous without cracks or discontinuity. The inset of Figure 1a corresponds to a higher magnification view of the top-surface image. It shows that the microstructure presented compact globular carbon-nanograin aggregates, similar looking to cauliflower, which in fact is known as “cauliflower type”. This cauliflower morphology is characteristic for films deposited by carbon thread evaporation, resulting from the atomic shadowing effect [64,65]. Figure 1b shows a well-adhered a-C film on the substrate, with a thickness of about 100 nm, confirming expectations for the carbon thread evaporation equipment.

**Figure 2:**
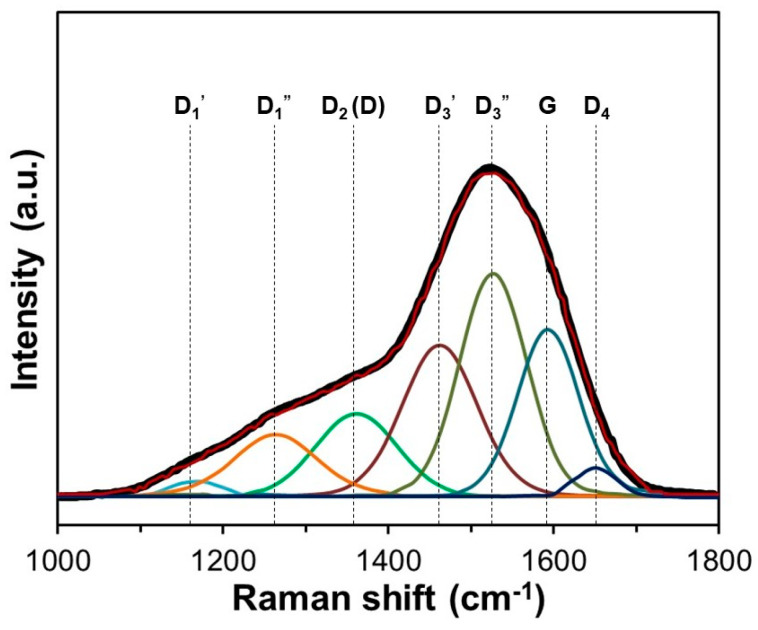
Raman spectra of the a-C film and its fitting using a 7-peak model.

Raman spectroscopy was employed to analyze the structural properties of the a-C film in the wave number range 1000–1800 cm^−1^, which corresponds to the one-phonon scattering region. Figure 2 exhibits the Raman spectra of the a-C film with the corresponding fitting using a seven-peak model. From low to high wave numbers, seven Raman modes were identified in the following order: D1′ (~1160 cm^−1^), D1″ (~1270 cm^−1^), D_2_ (D) mode (1360 cm^−1^), D3′ (~1470 cm^−1^), D3″ (~1530 cm^−1^), G mode (~1590 cm^−1^), and D4 mode (~1640 cm^−1^). Firstly, the D1′ mode (~1160 cm^−1^) was the result of a combination of vibrations due to the chain stretching composed of vinyl groups, C-H wagging modes, heteroatoms, and *sp*^2^ atoms found in defects and in an amorphous phase, and polyene and polyyne fragments in the a-C structure [66]. The D1″ mode (~1270 cm^−1^) may be associated with the characteristics of defects that are present in each type of carbon, which include point defects; stacking faults; and curved, edge, and twisted planes, among others [67]. The D_2_ (D) mode (~1360 cm^−1^) is attributed to a vibration of the A_1g_ symmetry, which is typical in disordered carbon structures [68]. The D3′ (~1470 cm^−1^) and D3″ (~1530 cm^−1^) modes are related to the presence of amorphous carbon [69]. The G mode (~1590 cm^−1^) is associated with in-plane vibrations of graphitic structure, characterized by E_2g_ symmetry, which involves an in-plane bond-stretching motion of C atom pairs [70]. Finally, the D4 mode (~1640 cm^−1^) is due to lattice vibrations, as well as vibrations from graphene layers present on the surface of graphite-like crystal [70].

**Figure 3:**
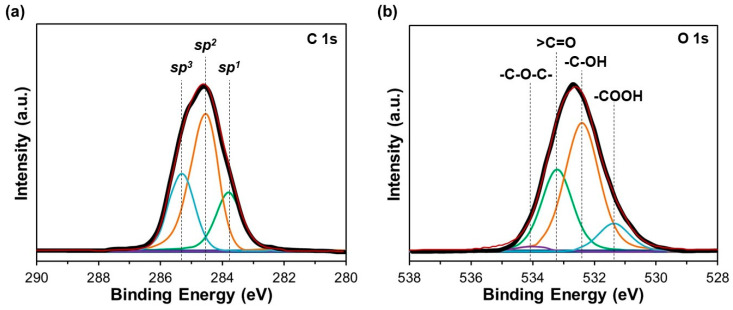
XPS spectra of (**a**) C1s and (**b**) O1s corresponding to the a-C film, indicating contents of *sp*^1^-, *sp*^2^-, and *sp*^3^-hybridized carbon atoms and oxygen-containing functional groups on the a-C’s surface.

**Figure 4:**
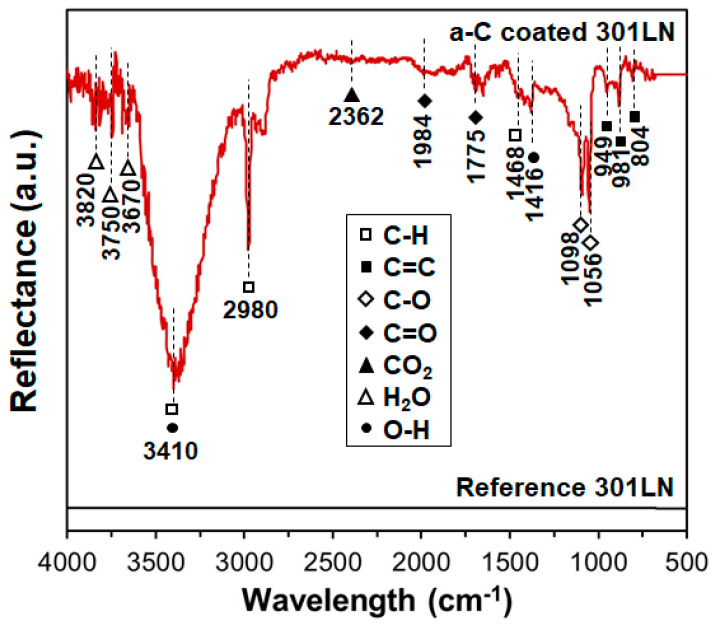
FTIR spectra of the surfaces of the reference and a-C-coated 301LN.

Figure 3 exhibits the XPS spectra of C1s and O1s for the a-C film. To determine the proportions of *sp*^1^-, *sp*^2^-, and *sp*^3^-hybridized carbon atoms, the C1s spectrum can be decomposed into three components corresponding to carbon clusters of *sp*^1^ (283.9 eV), *sp*^2^ (284.6 eV), and *sp*^3^ (285.3 eV) on the a-C’s surface (Figure 3a). On the other hand, as shown in Figure 3b, the O1s spectrum can be decomposed into four constituents associated with the following oxygen-containing functional groups: carboxyl -COOH (531.3 eV), hydroxyl -C-OH (532.3 eV), carbonyl >C=O (533.2 eV), and ether -C-O-C- (534.1 eV). The values found for the integral intensities indicate the following proportions of hybridized carbon atoms: 21% for *sp*^1^, 46% for *sp*^2^, and 33% for *sp*^3^. Therefore, these results evidence the significant formation of *sp*^2^-hybridized carbon clusters on the a-C film. In the case of the oxygen-containing functional groups, the percentages were -COOH (11%), -C-OH (51%), >C=O (36%), and -C-O-C- (2%), which are in good agreement with the FTIR spectra (Figure 4).

### 3.2. Analysis of Oxide Scales

**Figure 5:**
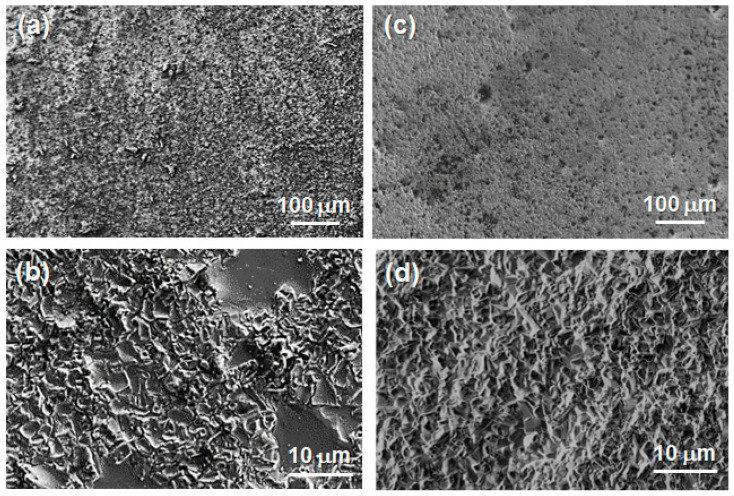
SEM images of the oxide layers that formed on (**a**,**b**) the reference and (**c**,**d**) a-C-coated 301LN samples after exposure to molten salt for 1000 h.

After the corrosion tests, the oxide scales formed on the reference and a-C-coated 301LN samples were analyzed to determine the effect of the a-C on corrosion mitigation for stainless steels. SEM images of the oxide layers that generated after exposure to molten salt for 1000 h are presented in Figure 5. Comparing the microstructures of the top surfaces of the oxide scales on both samples, remarkable differences can be observed. In Figure 5a,b, the reference 301LN sample exhibits large regions with a high density of agglomerates, as well as the presence of peel-off zones, which have a high content of Cr according to the EDX analyses. This suggests an oxide scale with a multilayer arrangement, in which the inner layer with a Cr-rich phase was covered with other LiFeO_2_-containing phases [71,72,73]. In contrast, the a-C-film-coated sample presents a more uniform aspect, considering the form and size of the crystals without peel-off (Figure 5c,d).

In terms of the corrosion behavior, the corrosion rate decreased from 0.33 ± 0.07 mm/year for the reference sample to 0.26 ± 0.08 mm/year for the a-C-coated 301LN, resulting in a decrease of about 20%, as estimated by the descaling analysis method. In addition, considering the peel-off observed in the reference sample, which did not take place for the a-C-coated 301LN, the extent of the corrosion of the coated sample may be assumed to have been reduced by more than 20%. This reduction in the corrosion rate using the a-C coating method is comparable to other alternative techniques used for this purpose in this type of energy storage system. For instance, the corrosion rates for the stainless steels AISI 301LN, AISI 310, and duplex 2205 in carbonate salts decreased by 20% [34], 45% [51], and 48% [33] by laser-texturing treatment compared with the untreated ones, respectively. Alternatively, doping nanoparticles (Al_2_O_3_ or SiO_2_) in molten carbonate salts significantly reduced the corrosion rate by ~50% compared with a base salt [43]. However, this method modifies the thermophysical properties of the molten salts. Thus, both the corrosion tests and microstructural analysis evidence that the incorporation of the a-C film stabilized the growth of the oxide scales, thus contributing to a more homogeneous oxidation of the surface and denser corrosion products, reducing the corrosion rate.

**Figure 6:**
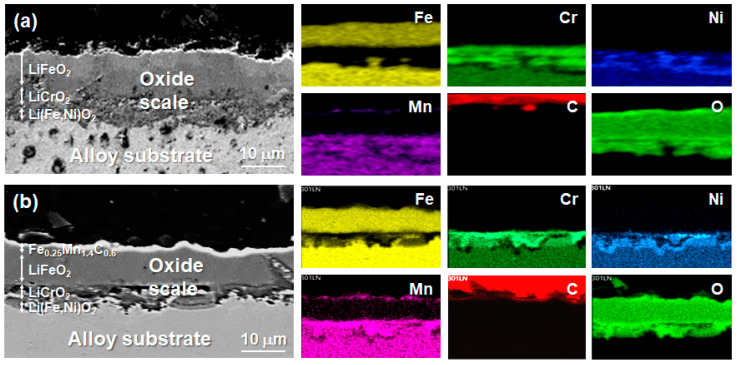
Cross-sectional SEM images and EDS mappings of the oxide scales formed on the (**a**) reference and (**b**) a-C-coated 301LN samples after 1000 h of exposure to molten salt.

Figure 6 displays cross-sectional SEM images and EDS mappings of the oxide layers that formed on the reference and a-C-coated 301LN samples after 1000 h of exposure to molten salt. For both, the oxide scales exhibited the characteristic distribution of corrosion products, resulting in a multilayer structure on the steel surface. For the oxide scales of the reference 301LN sample, the outer layer was enriched in Fe, while the inner layer had higher concentrations of Cr and Ni. Furthermore, the oxide scales that formed on the a-C-coated surface presented enrichments in C, Fe, and Mn, thus forming carbide of Fe and Mn (Fe_0.25_Mn_1.4_C_0.6_). The presence of carbides in the oxide layers could significantly contribute to improving the corrosion resistance of the a-C-coated sample, as it is well-known the positive role of these carbides in acting as a barrier to mitigate the corrosion process.

**Figure 7:**
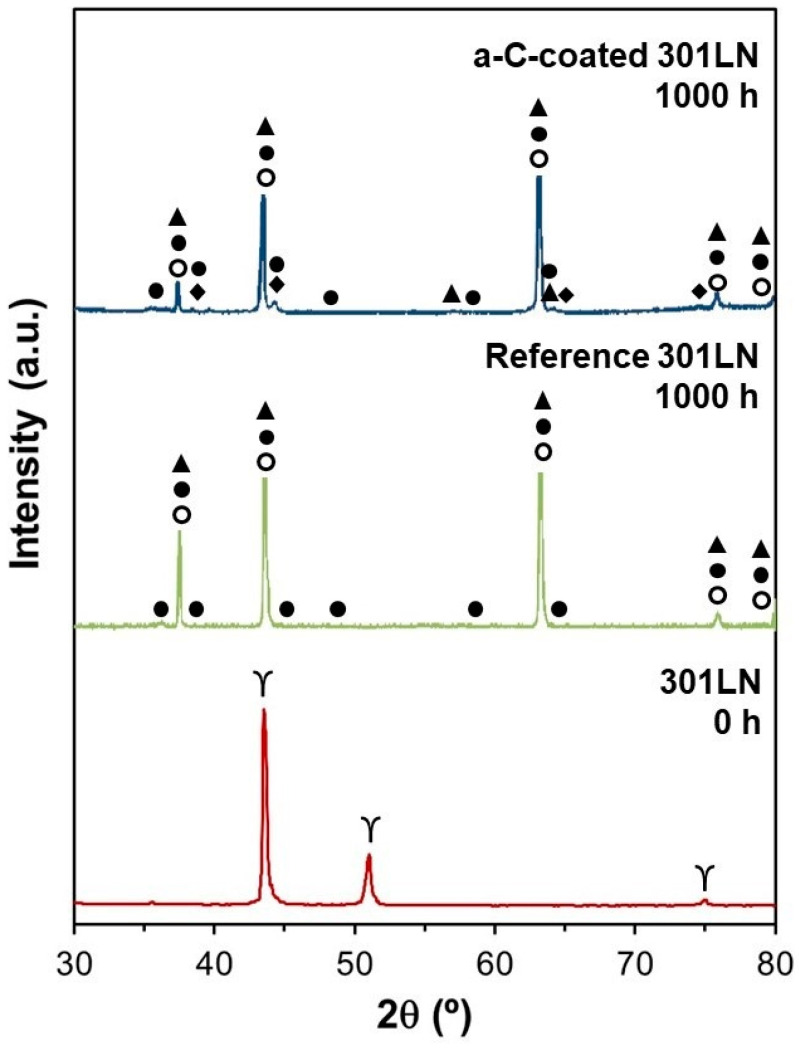
XRD spectra of top surfaces of the pristine, reference, and a-C-coated 301LN after 1000 h of exposure to molten salt. The symbols denote the following phases: γ—austenite (metallic substrate); ▲—LiCrO_2_; ●—Li(Fe,Ni)O_2_; ○—LiFeO_2_; ♦—Fe_0.25_Mn_1.4_C_0.6_.

Figure 7 displays the XRD spectra of the pristine, reference, and a-C-coated 301LN after 1000 h of exposure to molten salt. For the oxide scales of the reference sample, LiFeO_2_, LiCrO_2_, and Li(Fe,Ni)O_2_ were detected using SEM-EDX and XRD (Figure 6 and Figure 7), which is in good agreement with previous works [50,74]. On the Fe-rich outer layer, mainly LiFeO_2_ was generated, while LiCrO_2_ and Li(Fe,Ni)O_2_ formed at the Cr/Ni-rich inner layer. LiFeO_2_ does not provide effective corrosion protection because of its porosity in the oxide layers. However, both LiCrO_2_ and Li(Fe,Ni)O_2_ present better performances as corrosion protection layers. In contrast, the oxide scale of the a-C-coated sample formed in molten salt included LiCrO_2_, Li(Fe,Ni)O_2_, and LiFeO_2_, as well as Fe_0.25_Mn_1.4_C_0.6_ at the outer layer, which was observed from the complementary analyses of the XRD and SEM-EDS results (Figure 6 and Figure 7). This suggests that the presence of the carbide reinforced the outer oxide layer, obstructing the cation diffusion (Cr, Fe, etc.) from the substrate. The cation concentrations in the molten salts, as determined by ICP-OES after the corrosion tests, confirm a significant difference in the dissolutions of Cr, Fe, and Mn for the reference and a-C-coated 301LN (Table 1). The higher contents of these elements upon the interaction of salt with the reference sample prove the dissolution of Cr and Mn and corroborate the peel-off of the oxide scales during the corrosion test (Figure 5). In addition, it is also in good agreement with the significant presence of C, Fe, and Mn at the top surface of the oxide scales in the a-C-coated 301LN, as observed by the cross-sectional SEM-EDS analysis (Figure 6). This evidences that Fe_0.25_Mn_1.4_C_0.6_ contributes significantly to improving the corrosion resistance of the coated sample, because it helped produce a uniform and dense growth of oxide scales compared with the non-coated sample. Thus, the a-C film on the 301LN surface exhibited a favorable chemical compatibility with the 301LN and molten salt, reducing the corrosion rate compared with the reference sample without the a-C film.

### 3.3. Mechanical Behavior of Oxide Scales

**Figure 8:**
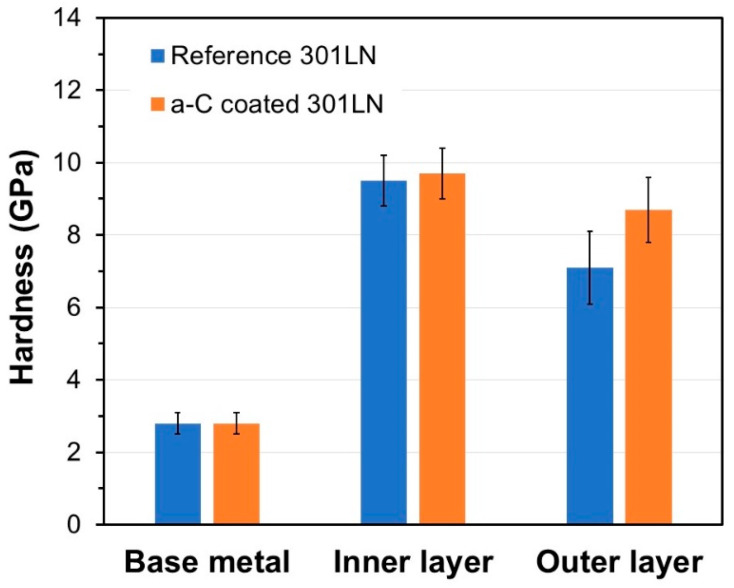
Hardness of the base metal and the inner and outer layers of the oxide scales for the reference and a-C-coated 301LN samples after 1000 h of exposure to molten salt.

The effect of the amorphous carbon film on the mechanical response of the oxide scales formed during the corrosion tests was investigated using the nanoindentation technique. Figure 8 presents the hardness values for the base metal and the inner and outer oxide layers for the reference and a-C-coated samples after 1000 h of exposure to molten salt. In both samples, the hardness of the 301LN sample was 2.8 ± 0.1 GPa. This value is quite similar to other 300 series stainless steels, such as AISI 310 (2.6 ± 0.1 GPa) [75], 316L (2.8 ± 0.1 GPa) [76,77], and 347 (2.4 ± 0.2 GPa) [78], materials commonly employed in CSP applications. Comparing the hardness of the inner oxide layer of the coated sample with the reference sample, no significant difference was observed. This is in good concordance with the dense corrosion products (LiCrO_2_ and Li(Fe,Ni)O_2_) of the inner layers for both samples. In contrast, the hardness of the outer oxide layer of the coated sample was higher than for the reference one, which presented mainly LiFeO_2_, which typically tends to generate porous oxide layers. This increase in the mechanical response was attributed to the formation of iron/manganese carbides generated at the top surface of the outer layer, therefore contributing to the generation of denser corrosion products at this oxide layer. A clear relationship is evident between the effectiveness of the protection provided by the oxide scales and their hardness and microstructure. Thus, the hardness values provide insight into the improvement in the quality of the scales formed with the deposition of the amorphous carbon film.

### 3.4. Mechanism Discussion

**Figure 9:**
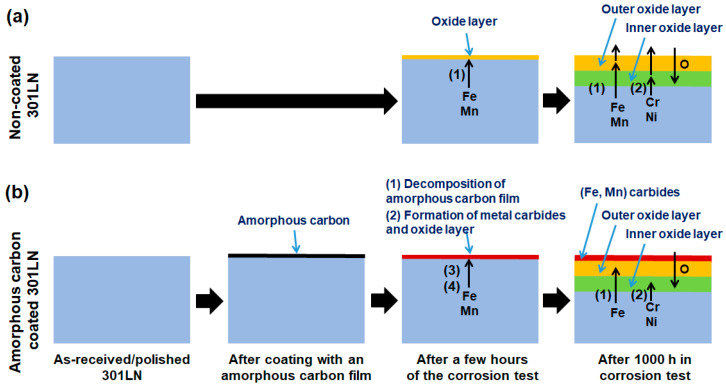
Mechanisms involved in the formation of oxide-scales on the surface of 301LN in molten carbonate salt, indicating the main phases and reactions that explain the effect of the amorphous carbon film on the reduction in corrosion of the (**a**) non-coated steel and (**b**) carbon-coated steel. The numbers (1), (2), (3) and (4) indicate the main reactions of the corrosion products.

The obtained results suggest that the amorphous film deposited on the 301LN inhibited corrosion in molten carbonate salt. To implement this anti-corrosion method for other structural steels used in CSPs, it is crucial to understand the formation mechanism of the protective layer. Figure 9 shows schemes of the corrosion process for the non-coated and a-C-coated 301LN samples. In the case of the non-coated steel (Figure 9a), the formation of LiFeO_2_ and LiCrO_2_ takes place through the well-known Reactions (1) and (2) [72,73]. From the cross-sectional SEM images and the chemical analysis of the molten carbonate salts, it becomes evident that LiFeO_2_ does not provide protection, and, therefore, LiCrO_2_ tends to dissolve in the molten carbonate salt.

(2) $${{Fe}_{2}O}_{3}+{{Li}_{2}CO}_{3}\to {2LiFeO}_{2}+{CO}_{2}$$

(3) $${{Cr}_{2}O}_{3}+{{Li}_{2}CO}_{3}\to {2LiCrO}_{2}+{CO}_{2}$$

For the a-C-coated 301LN sample, after the first hours of the corrosion test, a protective layer containing iron/manganese carbide formed through Reactions (3) and (4) [79,80], as follows:

(4) $$(Fe,Mn)+C\to {\left(Fe,Mn\right)}_{x}C$$

(5) $${5{Fe}_{2}O}_{3}+{{28Mn}_{2}O}_{3}+C\to {{40Fe}_{0.25}Mn}_{1.4}C_{0.6}+{CO}_{2}$$

Afterward, during the next 1000 h of the corrosion test, the protective layer of carbides was consolidated, which contributed to the growth of dense oxide layers (Figure 6 and Figure 7). This chemically and mechanically stabilized the oxide scales (Figure 8), preventing both their peel-off (Figure 5) and the dissolution of the chromium-containing oxides (Figure 6) and, therefore, reducing the corrosion rate.

## 4. Conclusions

The use of an amorphous carbon (a-C) film to coat stainless steel by the carbon thread evaporation technique was investigated, for the first time, as a new surface modification method to mitigate corrosion in molten carbonate salt. To validate this approach, non-coated and coated samples were immersed in carbonate molten salt at 600 °C for 1000 h. The main conclusions can be summarized as follows:

- The deposition of a uniform and defect-free a-C film on the 301LN’s surface was successfully achieved through the carbon thread evaporation technique. Analyses show that the a-C film was dense and uniform, presenting an amorphous structure formed mainly of *sp*^2^-hybridized carbon clusters with the presence of oxygen-containing functional groups;
- Coating the 301LN substrates with a thin a-C film enhanced the anti-corrosion protection, reducing the corrosion rate by more than 20%;
- The mechanism of the corrosion mitigation is mainly attributed to the generation of iron and manganese carbide at the top surface of the oxide scales. During the corrosion test, the a-C film decomposed, promoting the generation of carbide layers, which contributed to the formation of denser corrosion products and chromium oxide layers, increasing the hardness of the outer oxide layer. This chemically and mechanically stabilized the oxide scales, minimizing the diffusion of Cr and Ni through them, which led to thinner and more protective scales. As a consequence, Li^+^ diffusion through the 301LN substrate, as well as peel-off of the oxide scales, was reduced.

This work evidences that the a-C film contributed to the stabilization of the oxide scales that formed on the 301LN substrate, thus enhancing their effectiveness as a protective barrier. Additional costs due to the film being applied in a low-vacuum atmosphere and limitations related to the size of the treated components should be considered when evaluating the feasibility of using a-C films as a corrosion mitigation strategy. Thus, this anti-corrosion approach should be implemented in small components for energy applications, such as next-generation CSP plants, molten-carbonate fuel cells, and power-to-heat-to-power systems, as a promising alternative to other anti-corrosion treatments.

## Figures and Tables

**Table 1:** Concentrations of Cr, Fe, and Mn in molten salts after the corrosion tests on the reference and a-C-coated 301LN as determined by ICP-OES. Other elements such as Ni were detected below the limit of quantification.

Element	Concentration (ppm) in the Reference 301LN	Concentration (ppm) in the a-C-Coated 301LN
Cr	1.69	0.11
Fe	2.83	0.22
Mn	0.37	0.13

## Data Availability

Data are contained within the article.

## References

[1] Liu M., Tay N.H.S., Bell S., Belusko M., Jacob R., Will G., Saman W., Bruno F. (2016). Review on concentrating solar power plants and new developments in high temperature thermal energy storage technologies. Renew. Sustain. Energy Rev..

[2] Lovegrove K., Csiro W.S. (2012). Introduction to concentrating solar power (CSP) technology. Concentrating Solar Power Technology.

[3] Lovegrove K., Pye J. (2012). Fundamental principles of concentrating solar power (CSP) systems. Concentrating Solar Power Technology.

[4] Liu M., Saman W., Bruno F. (2012). Review on storage materials and thermal performance enhancement techniques for high temperature phase change thermal storage systems. Renew. Sustain. Energy Rev..

[5] Prieto C., Ruiz-Cabañas J., Madina V., Fernández A.I., Cabeza L.F. (2022). Lessons learned from corrosion of materials with molten salts during molten salt tank preheating. Sol. Energy Mater. Sol. Cells.

[6] Cabeza L.F., Gutierrez A., Barreneche C., Ushak S., Fernández Á.G., Fernádez A.I., Grágeda M. (2015). Lithium in thermal energy storage: A state-of-the-art review. Renew. Sustain. Energy Rev..

[7] Bauer T., Pfleger N., Laing D., Steinmann W.D., Eck M., Kaesche S. (2013). High-Temperature Molten Salts for Solar Power Application. Molten Salts Chemistry.

[8] Reddy R.G. (2011). Molten salts: Thermal energy storage and heat transfer media. J. Phase Equilibria Diffus..

[9] Parrado C., Marzo A., Fuentealba E., Fernández A.G. (2016). 2050 LCOE improvement using new molten salts for thermal energy storage in CSP plants. Renew. Sustain. Energy Rev..

[10] Ruiz-Cabañas F.J., Prieto C., Madina V., Fernández A.I., Cabeza L.F. (2017). Materials selection for thermal energy storage systems in parabolic trough collector solar facilities using high chloride content nitrate salts. Sol. Energy Mater. Sol. Cells.

[11] Ding W., Bauer T. (2021). Progress in Research and Development of Molten Chloride Salt Technology for Next Generation Concentrated Solar Power Plants. Engineering.

[12] Sarvghad M., Maher S.D., Collard D., Tassan M., Will G., Steinberg T.A. (2018). Materials compatibility for the next generation of Concentrated Solar Power plants. Energy Storage Mater..

[13] Gomez J.C. (2011). High-Temperature Phase Change Materials (PCM) Candidates for Thermal Energy Storage (TES) Applications. http://www.osti.gov/bridge.

[14] Kenisarin M.M. (2010). High-temperature phase change materials for thermal energy storage. Renew. Sustain. Energy Rev..

[15] Myers P.D., Goswami D.Y. (2016). Thermal energy storage using chloride salts and their eutectics. Appl. Therm. Eng..

[16] Fernández A.G., Cabeza L.F. (2020). Corrosion evaluation of eutectic chloride molten salt for new generation of CSP plants. Part 1: Thermal treatment assessment. J. Energy Storage.

[17] Fernández A.G., Cabeza L.F. (2020). Corrosion evaluation of eutectic chloride molten salt for new generation of CSP plants. Part 2: Materials screening performance. J. Energy Storage.

[18] Mehos M., Turchi C., Vidal J., Wagner M., Ma Z., Ho C., Kolb W., Andraka C., Kruizenga A. (2017). Concentrating Solar Power Gen3 Demonstration Roadmap.

[19] Turchi C.S., Vidal J., Bauer M. (2018). Molten salt power towers operating at 600–650 °C: Salt selection and cost benefits. Sol. Energy.

[20] Nunes V.M.B., Lourenço M.J.V., Santos F.J.V., de Castro C.A.N. (2019). Molten alkali carbonates as alternative engineering fluids for high temperature applications. Appl. Energy.

[21] Fereres S., Prieto C., Giménez-Gavarrell P., Rodríguez A., Sánchez-Jiménez P.E., Pérez-Maqueda L.A. (2018). Molten carbonate salts for advanced solar thermal energy power plants: Cover gas effect on fluid thermal stability. Sol. Energy Mater. Sol. Cells.

[22] Prieto C., Fereres S., Ruiz-Cabañas F.J., Rodriguez-Sanchez A., Montero C. (2020). Carbonate molten salt solar thermal pilot facility: Plant design, commissioning and operation up to 700 °C. Renew. Energy.

[23] Sarvghad M., Steinberg T.A., Will G. (2017). Corrosion of steel alloys in eutectic NaCl + Na_2_CO_3_ at 700 °C and Li_2_CO_3_ + K_2_CO_3_ + Na_2_CO_3_ at 450 °C for thermal energy storage. Sol. Energy Mater. Sol. Cells.

[24] Sah S.P. (2020). Corrosion of 304 stainless steel in carbonates melt—A state of enhanced dissolution of corrosion products. Corros. Sci..

[25] Morales M., Gordon S., Fernández-Arana Ó., García-Marro F., Mateo A., Llanes L., Fargas G. (2022). Duplex Stainless Steels for Thermal Energy Storage: Characterization of Oxide Scales Formed in Carbonate Salts at 500 °C. Metals.

[26] Morales M., Cabezas L., Castro-Alloca M., Fargas G., Llanes L., Mateo A. (2022). Corrosion Evaluation of Austenitic and Duplex Stainless Steels in Molten Carbonate Salts at 600 °C for Thermal Energy Storage. Metals.

[27] Sarvghad M., Will G., Steinberg T.A. (2017). Corrosion of Inconel 601 in molten salts for thermal energy storage. Sol. Energy Mater. Sol. Cells.

[28] de Miguel M.T., Encinas-Sánchez V., Lasanta M.I., García-Martín G., Pérez F.J. (2016). Corrosion resistance of HR3C to a carbonate molten salt for energy storage applications in CSP plants. Sol. Energy Mater. Sol. Cells.

[29] Biedenkopf P., Bischoff M.M., Wochner T. (2000). Corrosion phenomena of alloys and electrode materials in molten carbonate fuel cells. Mater. Corros..

[30] Rouillard F., Charten F., Moine G. (2011). Corrosion Behavior of Different Metallic Materials in Supercritical Carbon Dioxide at 550 °C and 250 Bars. Corrosion.

[31] Aljaerani H.A., Samykano M., Saidur R., Pandey A.K., Kadirgama K. (2021). Nanoparticles as molten salts thermophysical properties enhancer for concentrated solar power: A critical review. J. Energy Storage.

[32] González-Fernández L., Anagnostopoulos A., Karkantonis T., Bondarchuk O., Dimov S., Chorążewski M., Ding Y., Grosu Y. (2022). Laser-induced carbonization of stainless steel as a corrosion mitigation strategy for high-temperature molten salts applications. J. Energy Storage.

[33] Morales M., Rezayat M., Mateo A. (2024). Enhancing the corrosion resistance of 2205 duplex stainless steel in molten carbonate salts by laser-surface texturing. J. Energy Storage.

[34] Rezayat M., Morales M., Moradi M., Mateo A. (2024). Laser wobbling surface texturing of AISI 301LN steel for enhancement of the corrosion resistance at high temperature. Opt. Laser Technol..

[35] Grosu Y., Nithiyanantham U., Zaki A., Faik A. (2018). A simple method for the inhibition of the corrosion of carbon steel by molten nitrate salt for thermal storage in concentrating solar power applications. NPJ Mater. Degrad..

[36] Grosu Y., Anagnostopoulos A., Navarro M.E., Ding Y., Faik A. (2020). Inhibiting hot corrosion of molten Li_2_CO_3_-Na_2_CO_3_-K_2_CO_3_ salt through graphitization of construction materials for concentrated solar power. Sol. Energy Mater. Sol. Cells.

[37] Raiman S.S., Mayes R.T., Kurley J.M., Parrish R., Vogli E. (2019). Amorphous and partially-amorphous metal coatings for corrosion resistance in molten chloride salt. Sol. Energy Mater. Sol. Cells.

[38] González-Fernández L., Serrano Á., Palomo E., Grosu Y. (2023). Nanoparticle-based anticorrosion coatings for molten salts applications. J. Energy Storage.

[39] Kondaiah P., Pitchumani R. (2021). Fractal textured surfaces for high temperature corrosion mitigation in molten salts. Sol. Energy Mater. Sol. Cells.

[40] Kondaiah P., Pitchumani R. (2022). Novel textured surfaces for superior corrosion mitigation in molten carbonate salts for concentrating solar power. Renew. Sustain. Energy Rev..

[41] Kondaiah P., Pitchumani R. (2022). Fractal coatings of Ni and NiYSZ for high-temperature corrosion mitigation in solar salt. Corros. Sci..

[42] Kondaiah P., Pitchumani R. (2024). Electrodeposited nickel coatings for exceptional corrosion mitigation in industrial grade molten chloride salts for concentrating solar power. Renew. Sustain. Energy Rev..

[43] Schuller M., Little F., Malik D., Betts M., Shao Q., Luo J., Zhong W., Shankar S., Padmanaban A. (2012). Molten Salt-Carbon Nanotube Thermal Energy Storage for Concentrating Solar Power Systems Final Report.

[44] Frangini S., Loreti S. (2007). The role of alkaline-earth additives on the molten carbonate corrosion of 316L stainless steel. Corros. Sci..

[45] Yang X., Jiang W., Ji C., Wang Q. (2022). Experimental study on heat storage and corrosion properties of ternary carbonate salt-based ZnO nanofluids for solar thermal energy storage. J. Therm. Anal. Calorim..

[46] Shin D., Banerjee D. (2010). Effects of silica nanoparticles on enhancing the specific heat capacity of carbonate salt eutectic (work in progress). Int. J. Struct. Chang. Solids.

[47] Wu Y.T., Ren N., Wang T., Ma C.F. (2011). Experimental study on optimized composition of mixed carbonate salt for sensible heat storage in solar thermal power plant. Sol. Energy.

[48] Tao Y.B., Lin C.H., He Y.L. (2015). Preparation and thermal properties characterization of carbonate salt/carbon nanomaterial composite phase change material. Energy Convers. Manag..

[49] Sang L., Liu T. (2017). The enhanced specific heat capacity of ternary carbonates nanofluids with different nanoparticles. Sol. Energy Mater. Sol. Cells.

[50] Grosu Y., Anagnostopoulos A., Balakin B., Krupanek J., Navarro M.E., González-Fernández L., Ding Y., Faik A. (2021). Nanofluids based on molten carbonate salts for high-temperature thermal energy storage: Thermophysical properties, stability, compatibility and life cycle analysis. Sol. Energy Mater. Sol. Cells.

[51] González-Fernández L., Anagnostopoulos A., Karkantonis T., Dimov S., Chorążewski M., Ding Y., Grosu Y. (2023). Laser-texturing of stainless steel as a corrosion mitigation strategy for high-temperature molten salts applications under dynamic conditions. Sol. Energy Mater. Sol. Cells.

[52] Agüero A., Audigié P., Rodríguez S., Encinas-Sánchez V., De Miguel M.T., Pérez F.J. (2018). Protective coatings for high temperature molten salt heat storage systems in solar concentration power plants. AIP Conf. Proc..

[53] Encinas-Sánchez V., Batuecas E., Macías-García A., Mayo C., Díaz R., Pérez F.J. (2018). Corrosion resistance of protective coatings against molten nitrate salts for thermal energy storage and their environmental impact in CSP technology. Sol. Energy.

[54] Porcayo-Calderon J., Sotelo-Mazon O., Salinas-Bravo V.M., Arrieta-Gonzalez C.D., Ramos-Hernandez J.J., Cuevas-Arteaga C. (2012). Electrochemical Performance of Ni20Cr Coatings Applied by Combustion Powder Spray in ZnCl_2_-KCl Molten Salts. Int. J. Electrochem. Sci..

[55] Gomez-Vidal J.C., Fernandez A.G., Tirawat R., Turchi C., Huddleston W. (2017). Corrosion resistance of alumina forming alloys against molten chlorides for energy production. II: Electrochemical impedance spectroscopy under thermal cycling conditions. Sol. Energy Mater. Sol. Cells.

[56] Gomez-Vidal J.C. (2017). Corrosion resistance of MCrAlX coatings in a molten chloride for thermal storage in concentrating solar power applications. NPJ Mater. Degrad..

[57] Piquot J., Nithiyanantham U., Grosu Y., Faik A. (2019). Spray-graphitization as a protection method against corrosion by molten nitrate salts and molten salts based nanofluids for thermal energy storage applications. Sol. Energy Mater. Sol. Cells.

[58] Gonzalez M., Nithiyanantham U., Carbó-Argibay E., Bondarchuk O., Grosu Y., Faik A. (2019). Graphitization as efficient inhibitor of the carbon steel corrosion by molten binary nitrate salt for thermal energy storage at concentrated solar power. Sol. Energy Mater. Sol. Cells.

[59] Scendo M., Staszewska-Samson K. (2019). Effect of Temperature on Anti-Corrosive Properties of Diamond-Like Carbon Coating on S355 Steel. Materials.

[60] Wei J., Guo P., Liu L., Li H., Li H., Wang S., Ke P., Saito H., Wang A. (2020). Corrosion resistance of amorphous carbon film in 3.5 wt% NaCl solution for marine application. Electrochim. Acta.

[61] G1 Standard Practice for Preparing, Cleaning, and Evaluating Corrosion Test Specimens. https://www.astm.org/g0001-03r17e01.html.

[62] Morales M., Rezayat M., Fargas G., Mateo A. (2024). Mitigating the corrosion of AISI 301LN steel in molten carbonate salts by doping with alumina nanoparticles for thermal energy storage applications. Sol. Energy Mater. Sol. Cells.

[63] Oliver W.C., Pharr G.M. (2004). Measurement of hardness and elastic modulus by instrumented indentation: Advances in understanding and refinements to methodology. J. Mater. Res..

[64] Zhang Y., Wang Y., Wang S., Wei W., Ge X., Zhu B., Shao J., Wang Y. (2020). Comparison of Carbon Thin Films with Low Secondary Electron Yield Deposited in Neon and Argon. Coatings.

[65] Zhao Q., Mou Z., Zhang B., Zhang X., Wang Z., Wang K., Gao K., Jia Q. (2020). Revealing the corrosion resistance of amorphous carbon films under heat shock via annealing. Diam. Relat. Mater..

[66] Vautard F., Ozcan S., Paulauskas F., Spruiell J.E., Meyer H., Lance M.J. (2012). Influence of the carbon fiber surface microstructure on the surface chemistry generated by a thermo-chemical surface treatment. Appl. Surf. Sci..

[67] Couzi M., Bruneel J.L., Talaga D., Bokobza L. (2016). A multi wavelength Raman scattering study of defective graphitic carbon materials: The first order Raman spectra revisited. Carbon.

[68] Shimodaira N., Masui A. (2002). Raman spectroscopic investigations of activated carbon materials. J. Appl. Phys..

[69] Dippel B., Jander H., Heintzenberg J. (1999). NIR FT Raman spectroscopic study of flame soot. Phys. Chem. Chem. Phys..

[70] Al-Jishi R., Dresselhaus G. (1982). Lattice-dynamical model for graphite. Phys. Rev. B.

[71] Tan L., Yang Y., Allen T.R. (2006). Oxidation behavior of iron-based alloy HCM12A exposed in supercritical water. Corros. Sci..

[72] Biedenkopf P., Spiegel M., Grabke H.J. (1997). The corrosion behavior of Fe-Cr alloys containing Co, Mn, and/or Ni and of a Co-base alloy in the presence of molten (Li,K)-carbonate. Mater. Corros..

[73] Dorcheh A.S., Durham R.N., Galetz M.C. (2016). Corrosion behavior of stainless and low-chromium steels and IN625 in molten nitrate salts at 600 °C. Sol. Energy Mater. Sol. Cells.

[74] Bell S., Steinberg T., Will G. (2019). Corrosion mechanisms in molten salt thermal energy storage for concentrating solar power. Renew. Sustain. Energy Rev..

[75] Hong Y., Zhou C., Zheng Y., Zhang L., Zheng J., Chen X. (2019). Dependence of strain rate on hydrogen-induced hardening of austenitic stainless steel investigated by nanoindentation. Int. J. Hydrogen Energy.

[76] England J., Uddin M.J., Ramirez-Cedillo E., Karunarathne D., Nasrazadani S., Golden T.D., Siller H.R. (2022). Nanoindentation Hardness and Corrosion Studies of Additively Manufactured 316L Stainless Steel. J. Mater. Eng. Perform..

[77] Zhang X., Yang D., Jia Y., Wang G. (2023). Microstructure and Nanoindentation Behavior of FeCoNiAlTi High-Entropy Alloy-Reinforced 316L Stainless Steel Composite Fabricated by Selective Laser Melting. Materials.

[78] Hajiannia I., Shamanian M., Kasiri M. (2013). Microstructure and mechanical properties of AISI 347 stainless steel/A335 low alloy steel dissimilar joint produced by gas tungsten arc welding. Mater. Des..

[79] Zhang Y., Schleich D.M. (1994). Preparation and Characterization of Iron Manganese Carbide by Reaction of the Oxides and Carbon in Nitrogen. J. Solid State Chem..

[80] Avakyan L., Manukyan A., Bogdan A., Gyulasaryan H., Coutinho J., Paramonova E., Sukharina G., Srabionyan V., Sharoyan E., Bugaev L. (2020). Synthesis and structural characterization of iron-cementite nanoparticles encapsulated in carbon matrix. J. Nanoparticle Res..

